# Proteomic Profile and *In Silico* Analysis in Metastatic Melanoma with and without BRAF Mutation

**DOI:** 10.1371/journal.pone.0112025

**Published:** 2014-12-01

**Authors:** Vito Michele Garrisi, Sabino Strippoli, Simona De Summa, Rosamaria Pinto, Antonella Perrone, Gabriella Guida, Amalia Azzariti, Michele Guida, Tommasi Stefania

**Affiliations:** 1 National Cancer Research Centre, Istituto Tumori “Giovanni Paolo II”, Bari, Italy; 2 Dept. of Basic Medical Sciences, Faculty of Medicine and Surgery, School of Medicine, University of Bari, Bari, Italy; Universidade de São Paulo, Brazil

## Abstract

**Introduction:**

Selective inhibitors of BRAF, vemurafenib and dabrafenib are the standard of care for metastatic melanoma patients with BRAF V600, while chemotherapy continued to be widely used in BRAF wild type patients.

**Materials and Methods:**

In order to discover novel candidate biomarkers predictive to treatment, serum of 39 metastatic melanoma vemurafenib (n = 19) or chemotherapy (n = 20) treated patients at baseline, at disease control and at progression, were analyzed using SELDI-TOF technology. In silico analysis was used to identify more significant peaks.

**Results:**

In patients with different BRAF status, we found 5 peptides significantly deregulated, with the down-regulation of the m/z 9176 peak strongly associated with BRAF mutation. At baseline as predictive biomarkers we identified 2 peptides - m/z 6411, 4075 – as significantly up-regulated in responders to chemotherapy and 4 peaks - m/z 5900, 12544, 49124 and 11724 - significantly up-regulated in longer vs shorter responders to vemurafenib. After response, 3 peptides (m/z 4658, 18639, and 9307) resulted significantly down regulated while 3 peptides m/z 9292, 7765 and 9176 appeared up-regulated respectively in chemotherapy and vemurafenib responder patients. In vemurafenib treated patients, 16 peaks appeared deregulated at progression compared to baseline time. In silico analysis identified proteins involved in invasiveness (SLAIN1) and resistance (ABCC12) as well as in the pathway of detoxification (NQO1) and apoptosis (RBM10, TOX3, MTEFD1, TSPO2). Proteins associated with the modulation of neuronal plasticity (RIN1) and regulatory activity factors of gene transcription (KLF17, ZBTB44) were also highlighted.

**Conclusion:**

Our exploratory study highlighted some factors that deserve to be further investigated in order to provide a framework for improving melanoma treatment management through the development of biomarkers which could act as the strongest surrogates of the key biological events in stage IV melanoma.

## Introduction

Melanoma is the fifth cause of cancer-related mortality worldwide [1]. Until 2011 only a few minimally effective treatments were available to treat metastatic melanoma (MM), leading to an overall survival of 6–8 months. More recently, significant advances in our understanding of the molecular biology of melanoma and the complex role of host immunity have opened the field of melanoma therapy to include new immunotherapeutic approaches to unlock the immune response and develop molecularly targeted agents [2], [3].

As known, about 50% of melanomas harbour mutations in the BRAF gene, mainly at codon 600 (BRAF V600), resulting in constitutive activation of the MAPK pathway [4]. The selective inhibitors of BRAF V600, vemurafenib and dabrafenib have shown major tumour responses in about 50% of patients, resulting in improved progression free (PFS) and overall survival (OS) in MM compared with chemotherapy [5], [6]. However, the majority of patients progress after 6–8 months due to several resistance mechanisms which are only partially understood.

The monoclonal antibody ipilimumab, which targets the immune checkpoint CTLA-4, has shown survival benefits both as first and second line therapy [7]. However, the response rate to this drug is about 15% and only a few patients obtain a very long control of the disease.

As the majority of patients progress after a few months with anti-BRAF drugs, and ipilimumab is approved in Italy for second line only, chemotherapy continues to play an important role in a considerable number of MM patients.

Innovative chemotherapy modalities and new chemotherapeutic agents are now available for these patients and for those carrying the BRAF gene wild type. Among these, abraxane, a solvent-free albumin-stabilized nanoparticle formulation of paclitaxel, showed a particular activity in phase II and phase III trials [8], [9]. Another promising strategy utilizes resistance-modulating drugs with alkylating agents such as procarbazine, dacarbazine and temozolomide (TMZ). It has been demonstrated that these drugs are able to modulate the DNA repair enzyme MGMT, which constitutes the primary mechanism of tumor resistance to alkylating agents such as nitrosureas and others [10], [11]. We previously reported for the first time the possibility to use sequential non-therapeutic low dose TMZ before full dose Fotemustine (FM), demonstrating the efficacy of this regimen in MM patients in the presence of a profile of low toxicity [12].

As both targeting agents and chemical drugs appear to benefit only certain subsets of patients, the identification of predictors of response is mandatory. Indeed several studies have been performed in order to detect novel candidate biomarkers suitable as prognostic tools.

One of the available strategies that facilitates the simultaneous analysis of a large number of factors in biological material is surface enhanced laser desorption ionization time of flight mass spectrometry (SELDI ToF MS). This platform is currently used to resolve proteins in biological specimens through binding to biochemically distinct protein chips. Moreover, by combining high throughput data with the ability to observe differentially expressed peptides, this technique has been applied in several studies concerning cancer biomarker discovery [13]–[17].

The purpose of the present study is to verify if this high-throughput technique is able to individuate novel candidate peptides useful as biomarkers to predict the response or resistance to treatment in two sets of patients treated with TMZ/FM and vemurafenib, respectively.

## Materials and Methods

### Patient features

Thirty-nine consecutive patients (23 female and 16 male), treatment naïve and with histologically confirmed stage IV MM, were enrolled in the study at the Oncology Department of the National Cancer Research Centre Istituto Tumori “Giovanni Paolo II” Bari (Italy). The period of accrual was from July 2010 to January 2013. Adjuvant immunotherapy and previous radiotherapy or locoregional treatments on non-target lesions were permitted.

Nineteen BRAF V600 patients (V600E in 15 patients and V600K in 4 patients) were treated with vemurafenib at the standard dose of 960 mg twice daily until progression or unacceptable toxicity. These patients were not studied for other genes because at the time of enrollment only BRAF gene mutation detection was a standard of care in the management of MM patients.

The remaining 20 patients were treated with chemotherapy according to our innovative schedule including oral TMZ administered at a single dose of 100 mg/m^2^ on days 1 and 2 followed by intravenous FM at a dose of 100 mg/m^2^ on day 2, 4 hours after TMZ [12]. Treatment was repeated every 3 weeks up to a maximum of 9 cycles. This group included 17 BRAFwt patients and 3 with the BRAF mutation (V600E). Due to a lack of commercial availability Vemurafenib was not used in these latter patients who were also not able to be enrolled in trials due to melanoma brain involvement.

Patients were eligible if they had measurable lesions; adequate renal, hepatic and bone marrow functions; an Eastern Cooperative Oncology Group (ECOG) performance status ≤2; a life expectancy of more than 12 weeks; and were 18 years or older. Patients underwent clinical and radiological evaluation with tumor assessments at baseline and then every 3 cycles (approximately every 12 weeks) in order to evaluate therapeutic effectiveness. At the same time points blood from all available patients was sampled for proteomic analysis.

Response Evaluation Criteria In Solid Tumors (RECIST) was used for efficacy assessment [18]. We defined disease control (DC) as partial response [PR] plus complete response [CR] plus stable disease [SD] for more than 24 weeks. We also assessed PFS, defined as the length of time during and after medical treatment during which the disease being treated does not get worse, and OS, which was defined as the length of time from the date of study entry until the patient's death or the end of the accrual period. The study has been approved by the Ethics Committee of Istituto Tumori “Giovanni Paolo II” of Bari as satellite project of the protocol GOIM 2904, EUDRACT code: 2009-016487-36. All patients signed the informed consent. The study was conducted in accordance with the international standards of good clinical practice. Patient characteristics are reported in Table 1.

**Table 1:** Patient characteristics.

	Overall	Patients treated with Vemurafenib group A	Patients treated with TMZ/FM group B
	n. 39	n. 19	n. 20
Median age	52 yrs (28–83)	54 yrs (28–83)	51 yrs (34–80)
Sex Male	23	12	11
Female	16	7	9
Stage IV			
M1a	7	4	3
M1b	9	4	5
M1c	23	11	12
Metastatic sites			
lung	21	11	10
liver	7	4	3
spleen	3	0	3
lymph nodes	23	11	12
soft tissue	15	10	5
bone	9	6	3
brain	7	3	4
other (gastric, adrenal gland)	2	0	2
BRAF gene			
V600E/K	22	19	3
wt	17	0	17
DCR	61%	84%	35%
Median PFS	5 (2÷29+)	5 (2÷26+)	3 (2÷29+)
Median OS	8 (2÷29+)	8 (4÷26+)	8 (2÷29+)

All samples were tested only for genetic evaluation of BRAF status.

### Proteomic profile: protein chip & data preparation

Blood specimens from the consecutive series of 39 patients (19 treated with vemurafenib [group A] and 20 treated with chemotherapy [group B]) were collected before starting systemic therapy (T1A [vemurafenib] T1B [chemotherapy]), and then at the time of tumor assessment. TR has been indicated as the blood sampled at established DC (TRA[vemurafenib] TRB [chemotherapy]), and TP as the samples collected at the time of progressive disease (TPA [vemurafenib] TPB [chemotherapy]). Among patients who achieved a DC, TRB was available in 5 out of 7 patients, while TRA was available in 11 out of 16 patients due to patient unavailability. Moreover, TPB samples were not analyzed in 5 of 19 patients who progressed as they were lost in follow-up, while TPA were not taken in 4 patients who had not progressed at the time of analysis 12 months or more after beginning vemurafenib.

In order to minimize variable effects due to sample collection, processing and storage temperature, all blood samples were managed in the same manner without any protocol amendment during the entire collection period. The collected blood was allowed to clot at room temperature for 1 h and centrifuged at 3000 rpm for 15 min. Serum samples were stored in aliquots at −80°C in the Institutional Biobank until further analysis. Copper coated IMAC 30 protein chips, which bind metal binding proteins, were used and prepared as previously described [14]–[17]. Spectra were generated in the mass to charge range of 1500 to 50,000 Da.

### In silico peptide identification

The Mascot Peptide Mass Fingerprint online tool (http://www.matrixscience.com/cgi/search _form.pl?FORMVER = 2&SEARCH = PMF) was used to identify proteins introducing m/z values of peaks, which resulted to be significantly differentially expressed in each comparison. The search was performed through the SwissProt database setting “Trypsin” as the proteolytic enzyme.

### Statistical analysis

Automatic peak detection was performed along the entire spectra using the Protein Chip Data Manager program (version 4.1; Bio-Rad Laboratories, Hercules CA) with the following settings: signal/noise ratio (first pass), 3; minimum peak threshold, 10%; cluster mass window, 0.3%; signal/noise ratio (second pass), 1.5.

Following peak detection and clustering, average peak intensities for all groups was calculated. Peptides with a m/z value scoring ±0.3% were considered identical. Expression Differences Mapping (EDM) analysis was applied in order to generate a cluster peaks list which describes how a singular peak is expressed all along the specimen spectrum. Subsequently P-values of the differential expression of each peak using the non-parametric Mann-Whitney-U test were calculated in the specific study groups (i.e. not responsive vs responsive). When two groups were compared, the ROC area was calculated.

After normalization, analyses were firstly conducted in order to discover differences in terms of protein expression between BRAFwt and BRAF V600 patients. Subsequently, the population was divided into two subsets with respect to treatment. With regard to group B, the clinical outcomes prediction was investigated by dividing the study population into two categories (patients who achieved DC and those who did not achieve DC). In group A, due to the high rate of DC (84%), we decided to investigate a response duration prediction by comparing patients with long response (PFS >6 months) vs patients with short response (PFS <6 months). In both groups (A and B) T1 *vs* TR and T1 *vs* TP were compared for individual patients.

To predict if a pathological feature such as M stage, site of disease (soft tissue, lymph nodes, viscera, brain), or a specific significantly expressed peptide could be considered an independent predictor variable, a multivariate analysis was performed. Age at diagnosis was also considered in the logistic regression. The Wald Chi-Square statistic, which tests the unique contribution of each predictor in the context of the other predictors, was evaluated. Data were considered significant when *P*<0.05.

Kaplan–Meier survival analyses were implemented to estimate the PFS functions after the samples were classified into risk groups according to the presence of deregulated peptide expression. Differences in survival risk between the two risk groups were assessed using the Mantel–Haenszel log-rank test. The larger area between the groups and its associated smaller P value from the Mantel–Haenszel log-rank test implicate a better classification model.

## Results

### Quality Control and Reproducibility

The quality control (QC) serum sample, obtained by 4 mixed serum samples from healthy control subjects (2 women and 2 men), was used. Both the coefficient of variation (CV) for intensity and mass/charge (m/z) were calculated based on duplicate sample testing. The intrachip and interchip CV for intensity were <5%, while the intrachip and interchip CV for m/z were <0.05%. These values indicated high reproducibility of spectra with SELDI-TOF MS.

**Figure 1:**
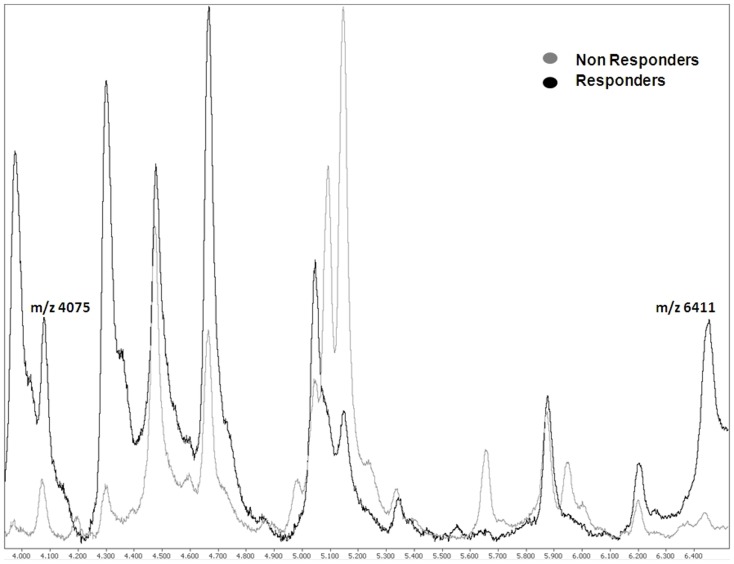
Representative example of fractionated serum protein profiles of two temozolomide/fotemustine treated patients (from responder and a non-responder patients).

The proteomic profile from serum samples was analyzed, and 60 protein peaks were detected between m/z 1500 and m/z 50000. A representative example of the SELDI-TOF MS protein profile is depicted in Fig. 1.

### Clinical results

Patients treated with chemotherapy received a median of 3 cycles (range 2÷9) of TMZ/FM. DC was observed in 7 patients (35%) with 5 PR and 2 SD. The median PFS was 3 months (range 2÷29+) and the median OS was 8 months (range 2÷29+).

In Vemurafenib-treated patients we documented a DC in 16 patients (84%), with 4 CR and 12 PR. The median PFS was 5 months (range 2÷20+) and the median OS was 8 months (range 4÷26+).

40% of all these patients underwent further lines of treatments with ipilimumab or subsequent chemotherapy. However, there was a low impact of subsequent treatments on median OS in both patient populations.

### Basal evaluation of proteomic profile according to BRAF status

Twenty-two out of 39 patients carried the V600 BRAF mutation, while 17 patients resulted to be BRAFwt. When comparing the two series before treatment, 3 peptides resulted significantly down-regulated while 2 peptides were up-regulated in BRAF-mutated patients (Table 2, Table S1). A multivariate analysis considering the risk factors M-stage, different site of metastasis and the 5 deregulated peaks showed a higher risk of BRAF mutation only when m/z 9176 was lower than the median value (OR:0,144, CI95%: 0,034÷0,633, p = 0.008) (Table 3).

**Table 2:** The 4 discriminating m/z peaks among BRAF V600E/K mutated and BRAFwt MM patients. m/z: mass-to-charge ratio; P was generated by peak comparison between BRAF mutated and wild type patients.

M/Z	P	Regulation	ROC Area	Intensity in BRAF mutated	Intensity in BRAF wt
9446	0,0148	down	0,715	6,182	8,43
9295	0,0217	up	0,715	16,709	8,296
1883	0,023	up	0,296	14,284	7,596
9176	0,025	down	0,704	6,9	8,22
4652	0,027	down	0,696	7,4	11,351

**Table 3:** Differential expression among vemurafenib treated patients; in all patients who achieved a response 3 peptides resulted significantly up-regulated at response evaluation: m/z 9292, 7765 and 9176.

M/Z	p-value	ROC Area	Intensity in T1A	Intensity in TRA	Regulation in TRA
9292	0.019	0.804	12.29	17.058	up
9176	0.033	0.727	6.08	6.57	up
7765	0.048	0.715	6.99	12.871	up

Only m/z 9292 resulted significantly up-regulated in short responders patients, while m/z 9176 and 7765 were significantly up-regulated in the long responder group.

### Evaluation of proteomic profile in patients treated with chemotherapy

The BRAFwt group plus 3 V600 BRAF carriers were treated with TMZ and FM as reported in M&M. In the specimens sampled before starting therapy (T1B) 2 significant peptides, m/z 6411 and 4075, were significantly up-regulated in the DC group with respect to the non-DC group (Table S2).

Multivariate proportional hazards analyses did not identify any independent risk factors with respect to therapy response among the selected peak clusters (m/z 6411 and 4075), age, M-stage and metastatic sites.

**Figure 2:**
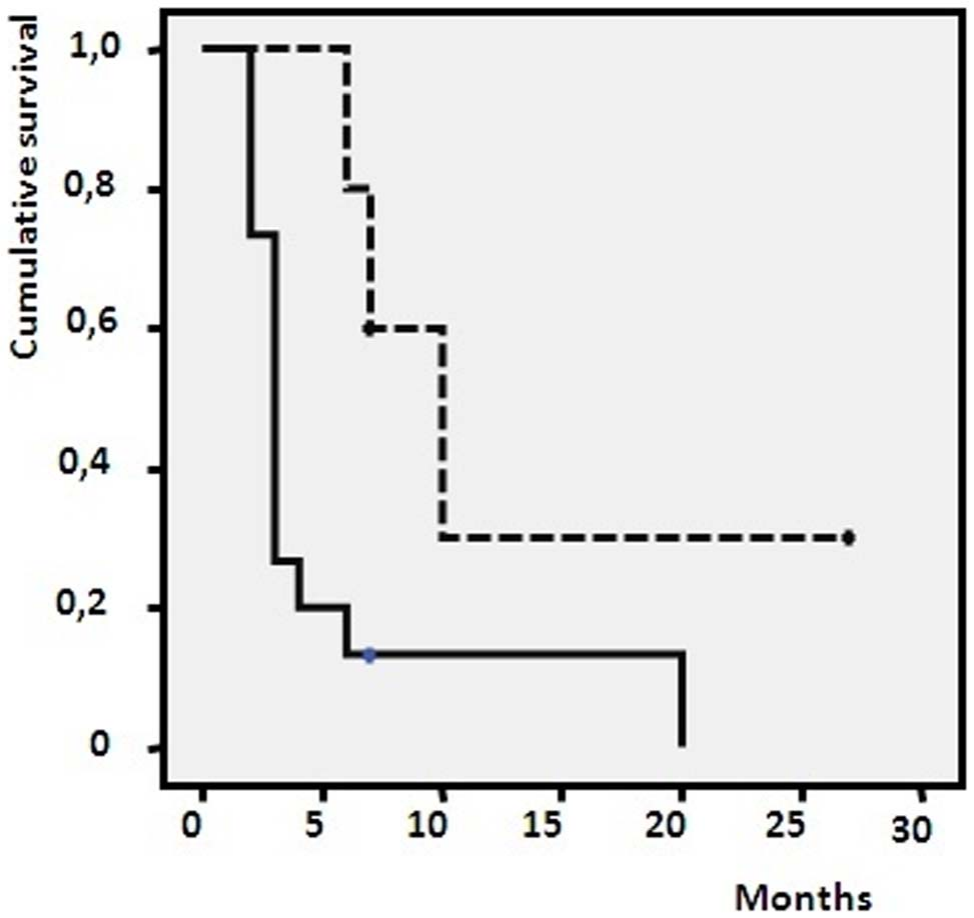
Progression free survival in TMZ/FM treated patients with respect to m/z 6411 and 4075 overexpression. The median value of expression has been considered as cutoff to discriminate peak overexpression. p = 0.024. **—**: patients with basal level of the 2 peaks; ---: patients with overexpression of both peaks.

Kaplan-Meyer analysis on the 2 peaks confirmed a significantly longer PFS in patients with up-regulation of both peptides m/z 6411 and 4075, p = 0,0024 (Figure 2).

For patients who achieved a DC, the comparison between T1B and TRB highlighted three down regulated peptides m/z (4658, 18639, and 9307) after therapeutic response (Table S3). However, Kaplan-Meyer analysis did not show a significant PFS improvement associated to the down-regulation of these peaks.

For patients who progressed at radiological and clinical evaluation, the comparison between T1B and TPB showed a similar peptide profiling (Table S4).

### Evaluation of proteomic profile in patients treated with Vemurafenib

**Figure 3:**
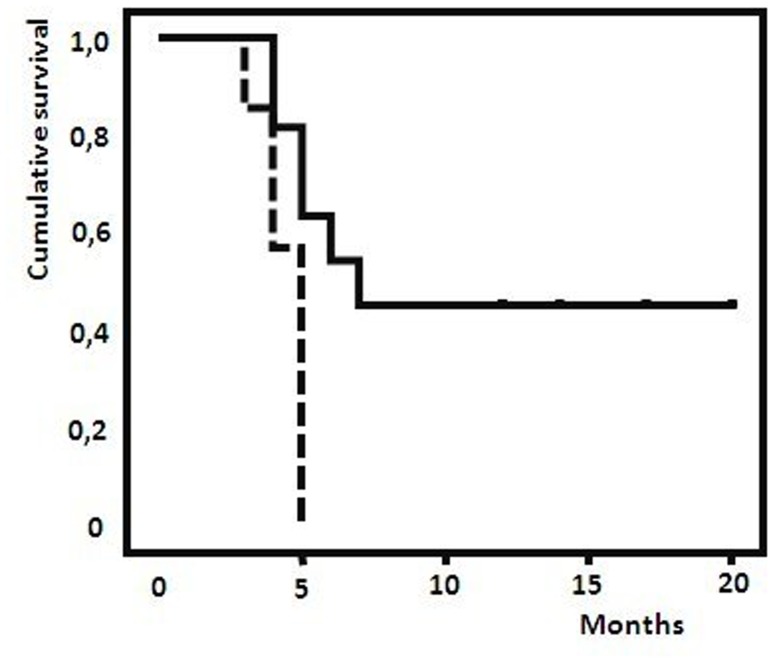
Kaplan-Meyer analysis which considered contemporary up-regulation of all 4 peaks (m/z 5900, 12544, 49124, 11724) with respect to progression free survival in patients treated with vemurafenib. p = 0.011. **—**: patients with basal level of the 4 peaks; ---: patients with overexpression of all 4 peaks.

Patients presenting a V600E/K mutation in the BRAF gene (n = 19) were treated with Vemurafenib as reported in M&M. All patients except 2 responded to therapy with a different time to progression. In the responder group, 11 patients had a PFS equal to or less than 6 months, and 6 patients had a PFS of more than 6 months. In the samples at baseline (T1A) of this set of patients, four peptides resulted to be significantly up-regulated in shorter vs longer responders: m/z 5900, 12544, 49124, 11724. These data were also confirmed by Kaplan-Meyer analysis which considered contemporary up-regulation of all 4 peaks with a significance of 0.011 (Figure 3).

In all patients who achieved a response when comparing T1A and TRA, 3 peptides resulted significantly up-regulated at response evaluation: m/z 9292, 7765 and 9176. Only m/z 9292 resulted significantly up-regulated in short responder patients, while m/z 9276 and 7765 were significantly up-regulated in the long responder group (Table 3, Table S5).

In patients for whom TPA samples were available, 16 peaks showed a significantly different expression with respect to basal time (T1A) or time of response to therapy (TRA). Moreover, evaluating only TPA compared to T1A, 21 peaks showed a significantly different expression, three of which (5335, 3238, 7765) presented higher intensity at progression (Table 4, Table S6).

**Table 4:** Differential expression among vemurafenib treated patients; in all patients who progressed 3 peptides resulted significantly up-regulated compared to basal time.

			INTENSITY (µa)	
PEAK (M/Z)	p-value	ROC	T1A	TPA
5335	0,0157	0,844	1,1175	10,251
3238	0,02086	0,844	2,1026	4,6736
7765	0,0274	0,844	6,9756	13,28346

### In silico peptide mass fingerprint

An in silico analysis was performed on peaks in order to discover possible associated biomarkers. We considered proteins showing a higher Mascot score and a higher percentage of sequence coverage. The 5 differently expressed peaks in BRAF V600 with respect to BRAFwt patients seem to be associated with proteins involved in invasiveness and resistance (Table 5). In the chemotherapy treated group, the 2 significantly upregulated peaks (m/z 6411, 4075) in responders could be associated with proteins involved in the pathway of detoxification (NQO1) and cell acidification (CA4, VATL), as reported in Table 6. In patients who achieved a DC with this treatment, the 3 downregulated peaks seemed to indicate proteins associated with modulation of neuronal plasticity (RIN1), transcription regulation (ZC3H6 and CNOT3) or calcium homeostasis (ASPH) (Table 6).

**Table 5:** Mascot search result for BRAF mutated vs BRAF wt patients.

Acrostic name	Description	Sequence coverage (%)	Score
SLAIN1	SLAIN motif-containing protein 1	20	38
GLIS2	Zinc finger protein	21	36
ABCC12	Multidrug resistance-associated protein 9	6	27
ATF6	Cyclic AMP-dependent transcription factor	12	18

**Table 6:** Mascot search result for BRAF wild-type patients.

BRAF wild-type patients				
	Acrostic name	Description	Sequence coverage (%)	Score
**Responder vs Non-Responder to TMZ/FE**	NQO1	NAD(P)H dehydrogenase [quinone] 1	21	28
	COMD5	COMM domain-containing protein - Hypertension-Related Calcium-Regulated Gene Protein	25	28
	CA4	Carbonic anhydrase 4	18	26
	VATL	V-type proton ATPase 16 kDa proteolipid subunit	41	26
	TM50A	Transmembrane protein 50A	37	26
**T1B vs TRB**	SGTA	Small glutamine-rich tetratricopeptide repeat-containing protein alpha	28	34
	CNOT3	CCR4-NOT transcription complex subunit 3	11	30
	RIN1	Ras and Rab interactor	11	27
	ZC3H6	Zinc finger CCCH domain-containing protein 6	7	25
	ASPH	Aspartyl/asparaginyl beta-hydroxylase	10	24

Among long responder patients treated with Vemurafenib, 4 upregulated peaks recognized factors with regulatory activity of gene transcription (KLF17 and TOX3 genes) and RNA alternative splicing (RBM10) (Table 7). Furthermore, the 3 response upregulated peaks (m/z 9292, 7765 and 9176) can be related to transcription regulatory factor (MTERFD1 in mitochondria), differentiation factors (CRX, TSPO2), transmembrane transporter (AQP11), factor within STAT3 pathway (IL7) and a protein with hydrolase activity (GGT7) (Table 7). Finally the most significantly different peaks of progression compared to baseline showed to be related to transcription regulatory factor (ZBTB44) as well as to proteins involved in endosome trafficking and chromosomal stability (PXK, TBC1D23) (Table 7).

**Table 7:** Mascot search result for BRAF mutated patients.

BRAF-mutated patients				
	Acrostic name	Description	Sequence coverage (%)	Score
**Longer vs shorter responder to Vemurafenib**	KLF17	Krueppel-like factor	31	40
	RBM10	RNA-binding protein	12	26
	TOX3	TOX high mobility group box family member 3	20	22
**T1A vs TRA**	MTEFD1	mTERF domain-containing protein 1, mitochondrial	20	33
	IL7R	Interleukin-7 receptor subunit alpha	18	28
	TSPO2	Translocator protein 2	50	28
	CRX	Cone-rod homeobox protein	31	24
	AQP11	Aquaporin-11	30	24
	GGT7	Gamma-glutamyltransferase 7	13	24
**T1A vs TPA**	TBC1D23	TBC1 domain family member	14	35
	PXK	PX domain-containing protein kinase-like protein	13	32
	PAPL	Iron/zinc purple acid phosphatase-like protein	16	30
	ZBTB44	Zinc finger and BTB domain-containing protein 44	12	28
	SPATA8	Spermatogenesis-associated protein	28	19
	FAM150A	Protein FAM150A	24	19
	PAIP2B	Polyadenylate-binding protein-interacting protein 2B	24	18

## Discussion

Therapeutic decisions are made selectively, tailoring therapy according to specific patient and tumor characteristics. Currently in MM, beyond “baseline” evaluation of BRAF mutations in the tumor sample to identify patients who are candidates to receive Vemurafenib, clinicians have no minimally invasive pharmacodynamic biomarkers in routine use to identify those patients most likely to benefit and to early monitor treatment efficacy. Serum biomarkers of melanoma are still awaited and the clinical significance of many evaluated peptides remains a matter of debate. Serum lactate dehydrogenase (LDH) represents the only marker which has been incorporated into the TNM classification as an independent and highly significant prognostic indicator [19], as shown in a multivariate analysis. However, notwithstanding its established use, this enzyme has a low sensitivity as a marker in MM [19] as it is also influenced by hemolysis and liver inflammatory injuries. Other serum molecules such as S100, C-Reactive Protein, Melanoma Inhibitory Activity (MIA), Galectin-3, melanin metabolites, cytokines, metalloproteinase, and adhesion proteins have been proposed as prognostic markers in melanoma, but to a less significant degree because of poorly-defined sensitivity/specificity [19], [20].

The aim of our exploratory study was to investigate serological tools in order to discover and develop relevant biomarkers, and to provide a framework for improving melanoma treatment management. We profiled serum peptides using the SELDI – TOF platform, an established proteomics approach. This serum analytic method, which combines higher throughput with the ability to observe differential protein expression levels, has already been applied to detect biomarkers in several cancer types [13]–[17]. To discover early diagnostic and prognostic predictors in stage III melanoma, similar serum proteomic analyses were carried out by Findeisen et al [21] and by Verdoliva V et al [22], who underlined the role of serum amyloid A, a2macroglobulin, Apolipoprotein-E and Apolipoprotein-A1. In this study we attempted in particular to uncover serum markers which could act as the strongest surrogates of key biological events in stage IV melanoma. Thus, owing to the biological heterogeneity of MM, we investigated the presence of different serum peptides in patients with diverse BRAF statuses. We found 5 peptides significantly deregulated, with the down-regulation of the m/z 9176 peak strongly associated with BRAF mutation. No previously defined melanoma biomarkers have been shown to be differently expressed with regards to MM molecular classification. Moreover, in order to attempt to give a preliminary identity to these peptides, an *in silico* analysis was performed by querying the Mascot search engine. Very interestingly, one of the identified peptide is a transcription factors trigger cancer epithelial-mesenchymal transition, SLAIN 1 whose expression was modulated by BRAF-mediated ERK activity in melanoma cells (23, 24). Another potential candidate marker is ATF6 which acts as sensor of the Endoplasmic Reticulum (ER)-induced Unfolded Protein Response (UPR) whose role in the different phases of tumor and melanoma growth is well known (25). Moreover in melanoma cell lines, the activation of ATF6 is also modulated by MEK/ERK signaling and thus conditioned by BRAF mutation status (26, 27). For the other 2 candidate proteins, ABBC2 and GLIS 2, lacks a clear evidence of relationship with melanoma or cancer.

Moreover, when comparing serum proteomic profiles at baseline in responder and non responder MM patients treated with chemotherapy or BRAF inhibitors, we were able to identify some markers correlated with response.

Even if there was a low DC rate, in a Kaplan-Meyer analysis we found that, in the presence of an up regulation of 2 predictors peaks, the PFS of chemotherapy-treated patients doubled. Interestingly, for patients treated with chemotherapy, one of the potential predictor molecules as identified by the Mascot engine was consistent with a potential chemotherapeutic sensitivity marker, NQO1 which controls redox cellular homeostasis and stabilizes the apoptosis regulator p53 towards degradation [28]. COMD5 and CA4, the two other proteins identified *in silico*, are considered markers of malignancy in cancer [29], [30] even if there is no evidence of their role in melanoma or in chemotherapy response. We were also able to assess the efficacy of chemotherapy, as the proteomic profile evidenced only in responder patients the down regulation of 3 peaks which could belong to molecules involved in the regulation of melanoma apoptosis machine like RIN1 [31]. Three other identified protein as markers of chemotherapy effectiveness are SGTA, CNOT3 and ASPH. All these protein play pivotal roles in various physiological functions, including cell proliferation, apoptosis, and metabolism and are just identified as cancer prognostic marker in various malignancies [32]–[34] even if no previous report referred to melanoma.

Equally, we identified 4 significantly up-regulated peaks in BRAF mutated patients correlated with a better response duration (more than 6 months), as shown in significant fashion by Kaplan-Meyer analysis. *In silico* predictions indicated that a metastatic suppressor gene such as KLF17 [35] and two cellular protein, RBM10 and TOX3 which are involved in proliferation and apoptosis of cancer cells [36], [37] could be the actors influencing the outcome of BRAF inhibitor therapy. Further markers of long response were identified by comparing the proteomic profile at baseline and at response-time. In this analysis we found a trend towards an up regulation of one peak in all BRAF inhibitor-treated patients, while two further peaks resulted up-regulated only in long responders. These latter molecules can be produced and secreted or shed into the bloodstream directly by melanoma cells or indirectly through their destruction. Furthermore, they can also derive from immune-mediated antitumor mechanisms triggered by the BRAF inhibitor, as suggested by Mascot identification of markers of autoimmunity like IL7RA as recently reported [38]–[39]. Other candidate markers of this set of patient's serum are apoptosis regulator like MTEFD1 and TSPO2 [40], [41] as well as a transcription factor, CRX whose expression and function is essential for growth of tumor cells with photoreceptor differentiation [42].

Finally in patient become resistant to BRAF inhibitor we highlighted the presence of 4 significantly deregulated peaks compared to baseline and response profile. This set of data could be very interesting due to the spasmodic attention which has been paid to the identification of the mechanisms inducing BRAF target therapy resistance, particularly in vivo. Among candidate molecules involved in these partially unknown events we identified by *in silico* analysis a transcription regulator, ZBTB44 which appears expressed in peripheral T-cell lymphoma [43] and interacts with SMAD pathway protein like SMURF2 mediating resistance to MAPK pathway inhibitors [43]–[45]. Other potential cellular component are PXK, implicated in epidermal growth factor receptor endosome trafficking and in hormone related cancer risk [46], [47], TBC1D23 which is involved in microsatellite instability cancers [48], FAM150A with unknown function in vivo but reported as hyper methylated in aggressive clear cell carcinomas [49]. For other identified molecules like PAPL and PAIP2B we were unable to find a clear link to melanoma or cancer. However SPATA8 mutation c.52G >A (p.Glu18Lys) is known to be linked with melanoma indicating its potential role in this disease.

In view of the increasing range of therapeutic options now available, an emerging challenge for clinicians is to establish a useful algorithm of sequential treatment for MM patients. Therefore, in the absence of sequential prospective studies, the choices of the correct agents, when to administer them and for how long are mostly guided empirically by clinical features of disease, such as bulk of disease and its evolutional speed, and patient characteristics such as performance status, age, presence of comorbidities [50]. In this direction, biomarkers could shed light on this matter. Our results, even if coming from an exploratory study on a limited number of patients, highlighted some factors that deserve to be further investigated because of their strict involvement in melanoma cell metabolism. Thus, to validate these preliminary results, a large prospective study in different cohorts of patients has been initiated.

## Supporting Information

Table S1 **Normalized data reporting cluster peaks' expression in BRAF mutated and BRAF wt patients.**
(CSV)

Table S2 **Normalized data reporting cluster peaks' expression in serum collected before treatment with TMZ in DC and non DC patients.**
(CSV)

Table S3 **Normalized data reporting cluster peaks' expression in serum of TMZ treated patients at moment of DC (TRB) compared to time before treatment (T1B).**
(CSV)

Table S4 **Normalized data reporting cluster peaks' expression in serum of TMZ treated patients at progression time (TPB) compared to time before treatment (T1B).**
(CSV)

Table S5 **Normalized data reporting cluster peaks' expression in serum of Vemurafenib treated patients at established disease control time compared to baseline.**
(CSV)

Table S6 **Normalized data reporting cluster peaks' expression in serum of Vemurafenib treated patients at progression compared to baseline.**
(CSV)
