# Correction: Proteomic Profile and *In Silico* Analysis in Metastatic Melanoma with and without BRAF Mutation

**DOI:** 10.1371/journal.pone.0122943

**Published:** 2015-03-30

**Authors:** The PLOS ONE Staff

The ninth author’s first and last names are incorrectly switched. The correct name is: Stefania Tommasi.
